# Complete Genome Sequence of *Lactobacillus rhamnosus* BFE5264, Isolated from Maasai Traditional Fermented Milk

**DOI:** 10.1128/genomeA.00563-17

**Published:** 2017-07-06

**Authors:** Sang-Haeng Choi, Yosep Ji, Soyoung Park, Julius Mathara, Wilhelm Holzapfel, Jihee Kang

**Affiliations:** a AtoGen Co. Ltd., Yuseong-gu, Daejeon, Republic of Korea; b Advanced Green Energy and Environment, Handong Global University, Pohang, Gyeongbuk, Republic of Korea; c Department of Food Science and Technology, Jomo Kenyatta University of Agriculture and Technology, Nairobi, Kenya

## Abstract

Here, we report the complete genome of *Lactobacillus rhamnosus* BFE5264, which was sequenced with the Pacific Biosciences RSII platform. The genome size is 3.01 Mb and includes 3,077 annotated coding sequences, including genes associated with the promotion of intestinal epithelial homeostasis through specific signaling pathways.

## GENOME ANNOUNCEMENT

*Lactobacillus rhamnosus* BFE5264 was isolated in Kenya from Maasai traditional fermented milk, kule naoto, in the early 2000s (1) and showed high survival under conditions simulating those of the upper gastrointestinal tract (2). This strain reduced cholesterol uptake in Caco-2 cells by downregulating the expression of NPC1L1, a critical factor for intestinal cholesterol absorption (3), and increasing cholesterol efflux in CHO-K1 cells by promoting the expression of ATP-binding cassette transporter A1 (ABCA1) and ATP-binding cassette transporter G1 (ABCG1) (4). These genes are the target genes for the cholesterol homeostasis regulators LXRα and LXRβ. The strain also showed cholesterol-lowering activity in an *in vivo* experiment using high-cholesterol-administered mouse model by modulating lipid metabolism-related factors (data not shown).

Several papers reported the relationship between gut microbiota and health, and especially probiotic strains of *Lactobacillus* have been suggested to support a healthy gut microbial environment (5). The administration of *L. rhamnosus* BFE5264 induced a change in the gut microbiota composition and some metabolites, such as short-chain fatty acids (SCFA), in an animal study (S. Park, Y. Ji, and W. Holzapfel, unpublished data). Further studies are needed to elaborate the underlying mechanisms of cholesterol regulation by* L. rhamnosus* BFE5264.

The complete genome sequence of *L. rhamnosus* BFE5264 was generated using the PacBio RS platform with single-molecule real-time (SMRT) sequencing at DNA Link (Seoul, South Korea). Annotations were performed by merging the results obtained from the Rapid Annotations using Subsystems Technology (RAST) server (6), Glimmer 3.02 modeling software, tRNAscan-SE 2.0, and RNAmmer 1.2. In addition, the contigs were searched against the KEGG, UniProt, and Clusters of Orthologous Groups (COG) databases to annotate the gene description. The G+C measurements (in mol%) were calculated using the genome sequences. *L. rhamnosus* strain BFE5264 consists of one circular chromosome (3,068,152 bp, GenBank accession no. CP014201) and one plasmid sequence (46,603 bp, accession no. CP014202). The chromosome contains a total of 3,077 predicted coding sequences (CDSs), with a G+C content of 46.8%. Sixty tRNA genes and 15 rRNA genes were identified. The plasmid contains a total of 62 CDSs, with a G+C content of 44%, and tRNA and rRNA were not identified. Open reading frames (ORFs) were found in the chromosome, with sizes ranging from 114 to 7,074 bp. Only 140 ORFs with lengths >2,000 bp were detected, with the majority being <1,500 bp. There were 335 subsystems represented in the chromosome, and we used this information to reconstruct the metabolic network (determined using the RAST server). A range of carbohydrate subsystem features was detected, including genes involved in disaccharide, oligosaccharide, and central carbohydrate metabolism. Among several protein metabolism features, genes for protein biosynthesis machinery, such as the large subunit (LSU) of the bacterial ribosome, were found. The most interesting findings were two genes associated with promote intestinal epithelial homeostasis through specific signaling pathways.

### Accession number(s).

This whole-genome sequence has been deposited at GenBank under the accession numbers CP014201 and CP014202.
